# Construction of KB@ZIF-8/PP Composite Separator for Lithium–Sulfur Batteries with Enhanced Electrochemical Performance

**DOI:** 10.3390/polym13234210

**Published:** 2021-12-01

**Authors:** Bingyi Ma, Xin Zhang, Xiaoqian Deng, Sheng Huang, Min Xiao, Shuanjin Wang, Dongmei Han, Yuezhong Meng

**Affiliations:** 1 School of Chemical Engineering and Technology, Sun Yat-sen Univeristy, Zhuhai 519082, China; maby@mail2.sysu.edu.cn (B.M.); zhangx349@mail2.sysu.edu.cn (X.Z.); 2111615014@mail2.gdut.edu.cn (X.D.); 2 The Key Laboratory of Low-Carbon Chemistry & Energy Conservation of Guangdong Province, State Key Laboratory of Optoelectronic Materials and Technologies, School of Materials Science and Engineering, Sun Yat-sen University, Guangzhou 510275, China; Huangsh47@mail.sysu.edu.cn (S.H.); stsxm@mail.sysu.edu.cn (M.X.); wangshj@mail.sysu.edu.cn (S.W.)

**Keywords:** lithium–sulfur battery, separator, shuttle effect, metal-organic framework, chemisorption

## Abstract

Lithium–sulfur batteries (LSBs) have attracted wide attention, but the shuttle effect of polysulfide hinders their further practical application. Herein, we develop a new strategy to construct a Ketjen black@zeolite imidazole framework-8/polypropylene composite separator. Such a separator consists of Ketjen black (KB), zeolite imidazole framework-8 (ZIF-8) and polypropylene (PP) with a low coating load of 0.06 mg cm^−2^ and is denoted as KB@ZIF-8/PP. KB@ZIF-8/PP can absorb polysulfides because of the Lewis acid-base interaction between ZIF-8 and polysulfides. This interaction can reduce the dissolution of polysulfides and suppress the shuttle effect, thereby enhancing the electrochemical performance of the battery. When tested at a current density of 0.1 C, an LSB with a KB@ZIF-8/PP separator exhibits low polarization and achieves a high initial capacity of 1235.6 mAh/g and a high capacity retention rate of 59.27% after 100 cycles.

## 1. Introduction

As a promising new generation of lithium secondary batteries, LSBs have the advantages of high theoretical specific capacity (1675 mAh/g) and high energy density (2600 Wh/kg) [1,2,3]. Moreover, sulfur in cathode materials is abundant in natural resources and environmentally friendly [4,5,6,7]. However, there are still some hurdles to overcome, such as lithium dendrites, electrode volume changes in the process of charge and discharge, poor conductivity of the cathode and particularly the shuttle effect [8,9,10]. During the working process of LSBs, the intermediate product polysulfides are easy to dissolve in the electrolyte, and react with the lithium anode through the separator, resulting in the corrosion of anode and serious loss of active materials and, what is worse, leading to the irreversible degradation of the battery capacity, which is called the shuttle effect [11,12,13]. An effective strategy to inhibit the shuttle effect is to modify the separator. Compared with the conventional PP separator that allows lithium polysulfide to travel between two electrodes, the modified separator can confine the polysulfides in the cathode side without affecting the normal circulation of lithium ions inside the battery [14,15]. Until now, lots of materials have been used in the field of separator modification, such as carbon host materials [16,17], polar metal compounds [18], conductive polymers [19,20] and metal-organic frameworks (MOFs) [21,22,23].

A MOF is a periodic porous structure formed by metal ions and organic ligands through metal–ligand complexation. Due to the large specific surface area, adjustable porosity, strong crystallinity and uniform morphology, MOFs have received growing attention [24,25]. As a Lewis acid-based material, MOFs can trap the polysulfides by Lewis acid-base interaction and suppress the shuttle effect to a certain extent. The porous structure also ensures the transport of lithium ions inside the lithium–sulfur battery [26,27,28,29]. Researchers have applied MOFs to separator modification and achieved considerable success. He et al. reported a Co_9_S_8_–Celgard separator which could capture polysulfides by physical and chemical effects [30]. The LSBs with a Co_9_S_8_–Celgard separator exhibited a high initial specific capacity of 1385 mAh/g at 0.1 C, high capacity of 1190 mAh/g after 200 cycles and low average capacity decay rate of 0.039% after 1000 cycles. Bai et al. developed a MOF@GO separator using Cu_3_(BTC)_2_ and graphene oxide, which could play a role in the physical capture and chemical absorption of polysulfide [31]. The initial capacity is 1126 mAh/g at 0.5 C, the capacity retention rate is up to 71% and the attenuation rate is only 0.019% after 1500 cycles. Hong et al. synthesized Ce-MOF/CNT material to modify the Celgard separator [21]. Because of the large specific surface area and numerous unsaturated metal sites, Ce-MOF can not only efficiently absorb polysulfides, but also promote the catalytic conversion of lithium polysulfides. With a sulfur loading of 2.5 mg cm^−2^, the batteries show a high initial specific capacity of 1021.8 mAh/g at 1 C and capacity retention of 838.8 mAh/g after 800 cycles.

In this work, high conductive KB@ZIF-8 composites were coated onto a PP separator to alleviate the shuttle effect. As a typical subclass of MOFs, ZIF-8 is composed of ZnN_4_ tetrahedral structural units formed by connecting Zn^2+^ with the N atom in the imidazole ring [32,33]. The unsaturated metal sites in ZIF-8 have a strong interaction with polysulfide, which can chemisorb lithium polysulfide. The large specific surface area helps to optimize the interface and reduce the loss of active materials [34,35,36]. One component of KB can not only improve conductivity, but also physically trap the intermediate product lithium polysulfide [37,38]. The battery with a KB@ZIF-8/PP separator delivered an excellent discharge specific capacity of 1236 mAh/g at 0.1 C. After 100 cycles, the specific capacity was 606 mAh/g and the capacity retention rate was 59.27%.

## 2. Materials and Methods

### 2.1. Synthesis of ZIF-8

405.0 mg of Zn(NO_3_)_2_·6H_2_O (AR, Macklin, Shanghai, China) and 263.0 mg of 2-methylimidazole (98%, Aikeda, Chengdu, China) were dissolved in 80 mL of methanol (99.5%, Macklin, Shanghai, China) and stirred for 15 min at room temperature. The mixed solution was allowed to stand for 48 h and then centrifuged at 5000 r/min. The obtained precipitate was washed thoroughly with ethanol (99.5%, Aladdin, Shanghai, China) three times and finally dried in vacuum at 60 °C for 24 h to obtain solid powder ZIF-8.

### 2.2. Preparation of KB@ZIF-8/PP Separator

To prepare a KB@ZIF-8/PP separator, ZIF-8 and KB (ECP-600JD, Guangdong, China) were mixed with a mass ratio of 1:4 and stirred mechanically in ethanol for 24 h, then ultrasonically dispersed for 20 min. After that, the homogeneous solution was coated onto a Celgard 2500 separator by vacuum filtration and then vacuum dried at 60 °C for 24 h. Finally, the KB@ZIF-8/PP separator was cut into discs with a diameter of 19 mm. For comparison, Ketjen black modified PP separators were prepared under the same condition too. The loading density of the KB@ZIF-8/PP separator and KB/PP separator was approximately 0.06 mg cm^−2^.

### 2.3. Electrode and Cell Preparation

Sulfur (99.95%, Aladdin, Shanghai, China), Super P (TIMCAL, Guangzhou, China) and PVDF (HSV900, Guangzhou, China) with a mass ratio of 7:2:1 were dissolved in NMP (AR, Aladdin, Shanghai, China) and grinded for 24 h to obtain a slurry. The slurry was evenly coated onto the aluminum foil and then vacuum dried at 60 °C for 24 h. Subsequently, the dried aluminum foil was punched into discs with a diameter of 12 mm. The mass loading of sulfur in the cathode was about 1.20 mg cm^−2^.

The sulfur cathode and lithium anode were assembled into a CR2025 coin cell in a glove box filled with Ar gas. The Celgard 2500 membrane was used as the separator. The electrolyte was composed of 1 M LiTFSI (98%, Aladdin, Shanghai, China) in a DME (99.5%, BLCR, Beijing, China):DOL (99.5%, BLCR, Beijing, China) (volume ratio was 1:1) mixture with a 1% mass fraction of lithium nitrate. The amount of electrolyte for each battery was 50 μL.

### 2.4. Material Characterization and Electrochemical Measurements

The morphology of the material was examined by scanning with an electron microscope (SEM, HITACHI S4800, Hitachi Ltd., Tokyo, Japan) and a transmission electron microscope (TEM, JSM-2010HR, JEOL Ltd., Tokyo, JapanFEI). The crystal structure was analyzed using a X-ray diffractometer (XRD, Dmax 2200, Rigaku Ltd., Tokyo, Japan) with Cu Kα radiation (λ = 0.15418 nm). The wettability of composite materials to electrolyte was evaluated by a contact angle tester (OCA15EC, Dataphysics, Stuttgart, Germany). The chemical bonds of samples were investigated by Fourier-transform infrared (FTIR, Analect Company, New York, NY, USA); X-ray photoelectron spectroscopy (XPS, ESCALAB250, Thermo Fisher Scientific Ltd., Waltham, MA, USA) was used to evaluate the chemical state of the sample surface. Electrochemical impedance spectroscopy (EIS, 0.1–100,000 Hz) and cyclic voltammetry (CV, 1.7–2.7 V, 0.01 mV/s) were tested by an electrochemical workstation (CHI 604E, Chenhua, Shanghai, China). A constant current charge and discharge measurement was performed on a LAND battery system (CT 200, Wuhanlanbo, Wuhan, China) at room temperature.

## 3. Results and Discussion

### 3.1. Characterization

**Figure 1:**
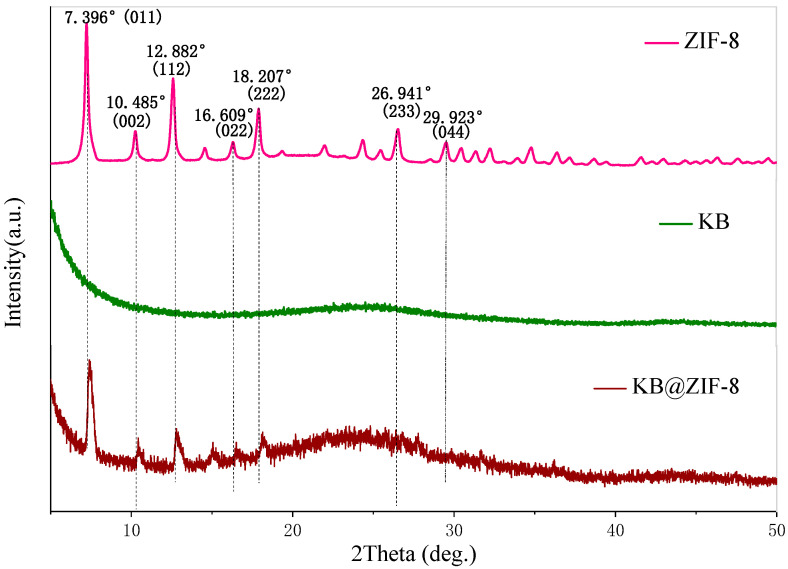
XRD patterns of ZIF-8, KB and KB@ZIF-8 composite.

Figure 1 presents the XRD patterns of ZIF-8, KB and KB@ZIF-8 composite material. The XRD pattern of ZIF-8 has strong diffraction peaks at 2θ = 7.396° (011), 10.485° (002), 12.882° (112), 16.609° (022), 18.207° (222), 26.941° (233) and 29.923° (044) in turn, indicating the successful synthesis of ZIF-8 with a typical rhombic dodecahedra structure [23,39]. The XRD pattern of KB@ZIF-8 is basically similar to that of KB. At the phase angles corresponding to the peaks of ZIF-8, there are also obvious diffraction peaks of KB@ZIF-8, illustrating that ZIF-8 and KB still maintain the integrity of their crystal structure during the mixing process.

**Figure 2:**
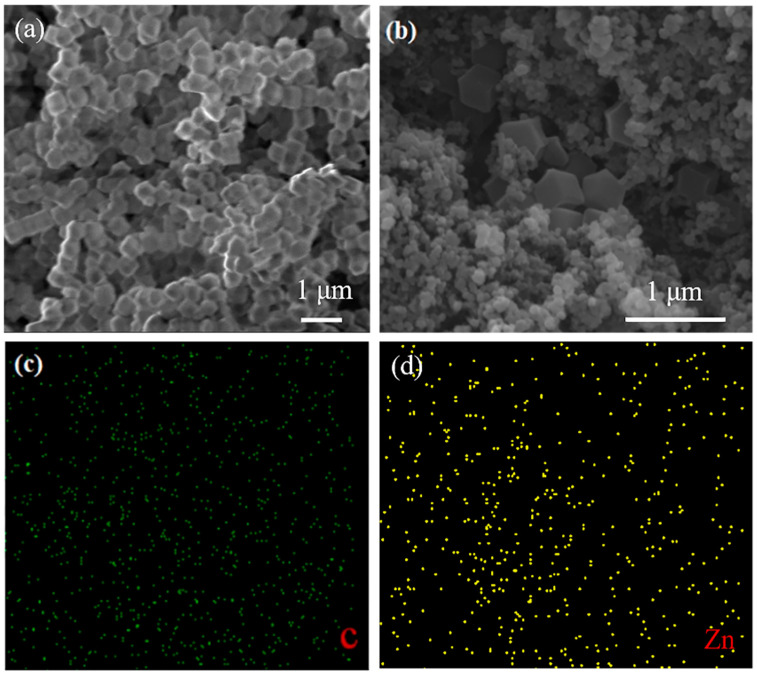
SEM image of (**a**) ZIF-8 and (**b**) KB@ZIF-8 composite, (**c**,**d**) mapping of C and Zn.

**Figure 3:**
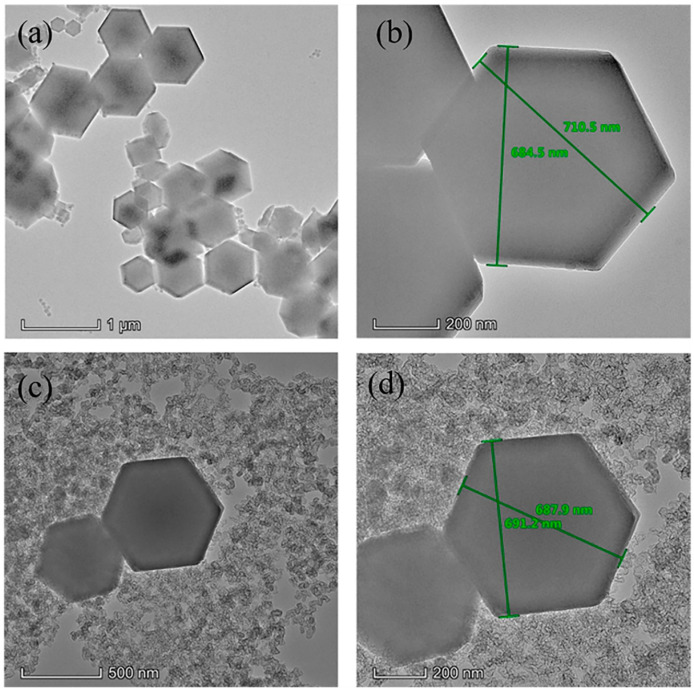
TEM image of (**a**,**b**) ZIF-8 and (**c**,**d**) KB@ZIF-8 composite.

**Figure 4:**
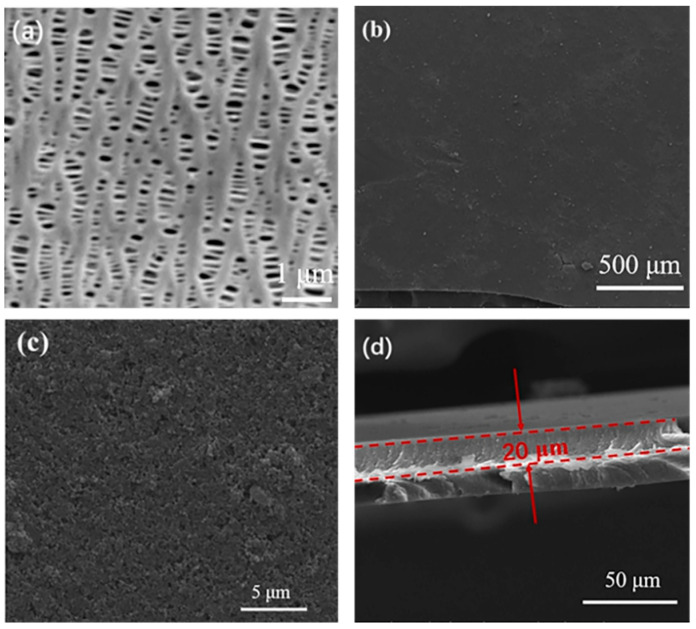
SEM image of (**a**) PP separator and (**b**–**d**) KB@ZIF-8/PP separator.

To further investigate the morphology, the prepared ZIF-8 and KB@ZIF-8 composite material were studied by both SEM and TEM. As presented in Figure 2a, the prepared ZIF-8 composite has a stacked crystal structure with hexagonal cross-sections. In Figure 2b, the particle size of the agglomerated KB is much smaller than that of ZIF-8. ZIF-8 is uniformly dispersed on the surface of KB. As shown in Figure 2c,d, the elemental mapping results of C and Zn show the uniform distribution of ZIF-8 in KB@ZIF-8 composites. Figure 3a,b also illustrate that the molecular size of prepared ZIF-8 is relatively uniform, with a particle size of approximately 700 nm. In Figure 3c,d, there are many KB particles distributed around ZIF-8. The composite of KB and Lewis acid-based ZIF-8 is expected to effectively alleviate the shuttle of polysulfide. The SEM image of a commercial PP separator is presented in Figure 4a. There are lots of close-packed pores on the surface, which can be used as the transmission channel for ions inside the battery. The surface and cross-sectional SEM images of KB@ZIF-8/PP are presented in Figure 4b–d, respectively. Some pores on the PP separator are covered by KB@ZIF-8 particles, while some pores are left for ions to transport. The surface of the modified separator is relatively uniform and flat and the thickness is about 20 μm.

**Figure 5:**
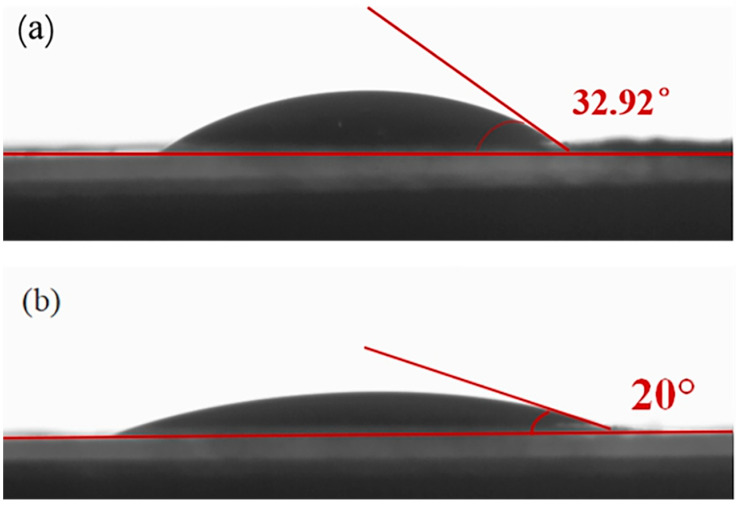
Contact angle of (**a**) PP separator and (**b**) KB@ZIF-8/PP separator.

The contact angle when it drops on the surface of the membrane is used to judge the wettability of the modified separator to the electrolyte of the LSB. As shown in Figure 5, the contact angle of the KB@ZIF-8/PP separator is 20°, smaller than that of the PP membrane, demonstrating that KB@ZIF-8 enhances the wettability of the separator with the electrolyte. It is beneficial to the interface electrochemical reaction and improves the electrochemical performance of the battery.

### 3.2. Electrochemical Performance

**Figure 6:**
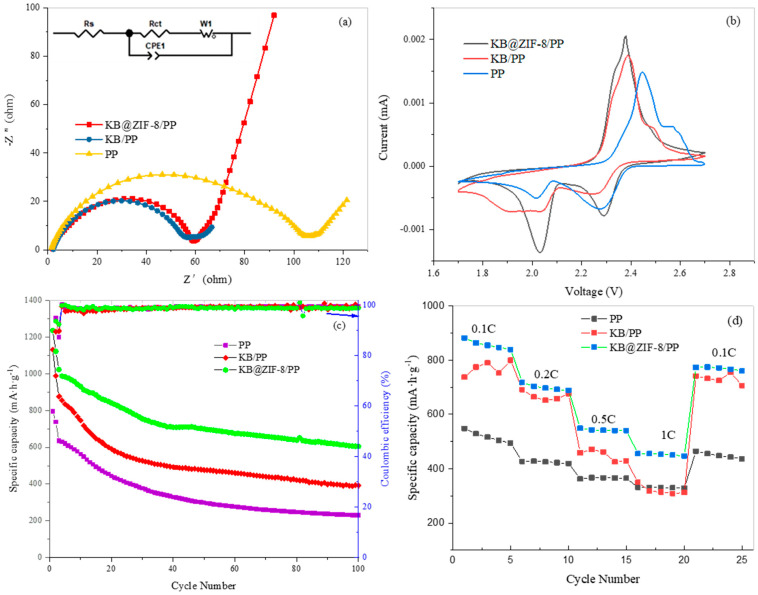
(**a**) EIS, (**b**) CV, (**c**) cycle performance for 100 cycles at 0.1 C, (**d**) rate performance of lithium–sulfur batteries with PP separator, KB/PP separator and KB@ZIF-8/PP separator.

EIS (electrochemical impedance spectroscopy) measurements were performed on LSBs with different separators (Figure 6a). AC impedance spectroscopy of an LSB is composed of a semicircle and linear zones. The semicircle in the high frequency region and the linear in the low frequency region are related to the charge-transfer resistance (R_ct_) and the diffusion of lithium ions in the electrode, respectively. In addition, the R_s_ is associated with the electron conduction between the electrolyte and Al collector [40]. The R_s_ of the KB@ZIF-8/PP,KB@PP and PP is 1.76 Ω, 2.213 Ω and 0.56855 Ω, respectively. The semicircle diameter of the KB@ZIF-8/PP separator is smaller than that of the PP separator, that is, the R_ct_ of KB@ZIF-8/PP is smaller. This confirms that the interfacial charge-transfer resistance of PP membrane can be reduced by the introduction of KB@ZIF-8. It may be ascribed to the enhanced wettability of the KB@ZIF-8/PP separator to the electrolyte, thus facilitating the charges transfer in the battery. The R_ct_ of KB@PP is the largest because of the excellent conductivity of KB. According to the fitted data, the R_ct_ of KB@ZIF-8/PP,KB@PP and PP is 58.17 Ω, 53.63 Ω and 101.3 Ω, respectively. The linear slope of KB@ZIF-8/PP increases slightly, implying that the diffusion resistance of lithium ions reduces. It is conducive to the transmission of lithium ions in the battery.

CV tests were performed to obtain information about the reversibility of electrode reaction and the reaction kinetics. The oxidation peak represents the conversion of polysulfide to S_8_. Two reduction peaks with high potential and low potential correspond to the reduction of S_8_ to Li_2_S_n_ (4 ≤ n ≤ 8) and Li_2_S/Li_2_S_2_, respectively. Figure 6b shows the cyclic voltammetry curves of coin cells assembled with a KB@ZIF-8/PP separator, KB/PP separator and PP separator. The PP cell exhibits an oxidation peak at around 2.45 V and two reduction peaks at 2.02 V and 2.28 V. The KB/PP cell displays an oxidation peak at around 2.39 V and two reduction peaks at about 2.04 V and 2.27 V. The decrease of potential difference manifests the reduction of electrochemical polarization in case KB is used. After the introduction of KB@ZIF-8, the voltage peaks are sharper and larger, implying that the battery capacity increases. The oxidation peak of the KB@ZIF-8/PP cell is 2.38 V, and the reduction peak is around 2.02 V and 2.30 V. The potential difference is further decreased, indicating the enhanced reversibility of the battery and the reduced electrochemical polarization. There are also two oxidation peaks in the charging process. The obvious peak at 2.3–2.4 V refers to the transition from insoluble short-chain sulfides to long-chain soluble polysulfides (Li_2_S/Li_2_S_2_→Li_2_S_n_ (4 ≦ n ≦ 8)). There is also a peak at around 2.5–2.6V, which corresponds to the process of converting long-chain polysulfides into sulfur (Li_2_S_n_→S_8_ (4 ≦ n ≦ 8)).

To study the effect of KB@ZIF-8 on the cycle performance of the battery, LSBs assembled with different separators were subjected to a charge-discharge test at a current density of 0.1 C, as shown in Figure 6c. The coulombic efficiency of all was close to 100%. The initial discharge specific capacity of the LSB with PP, KB/PP and KB@ZIF-8/PP separator was 795.9 mAh/g, 1130.3 mAh/g and 1235.6 mAh/g, in their turns. After 100 cycles, the specific capacity of the battery with KB@ZIF-8/PP decreased to 605.9 mAh g^−1^, but it was higher than that of KB/PP (390.6 mAh/g) and PP (230.2 mAh/g). The capacity retention rate of the battery with KB@ZIF-8/PP was 59.27%, which is higher than that of KB/PP (44.56%) and PP (36.28%). To compare, results in previously reported works were 56.78% at 0.5 C (500 cycles) [41], 40% at 1 C (1000 cycles) [42], 45.11% at 1 C (500 cycles) [43], 48.30% at 0.5 C (500 cycles) [44] and 57.32% at 0.5 C (300 cycles) [27]. The above results can be ascribed to the physical adsorption of KB and chemisorption of ZIF-8 to polysulfide. The synergy of ZIF-8 and KB helps to restrict the polysulfide on the cathode and therefore improve the utilization of the active substance and mitigate the shuttle effect.

To evaluate the rate performance of cells, the LSBs with different separators were analyzed at different densities. The current density increased from 0.1 C to 1 C, and, finally, returned to 0.1 C. As exhibited in Figure 6d, the discharge specific capacity of KB@ZIF-8/PP is higher than that of KB/PP and PP at the same rate. As the current increases, the specific capacity decreases. The specific capacity of KB@ZIF-8/PP decays more slowly compared with that of KB/PP and PP. Especially at 0.5 C and 1 C, its discharge capacity is significantly higher. After 20 cycles, when the current density is reduced to 0.1 C again, the specific discharge capacity can be restored to a high value, revealing the good reversibility of KB@ZIF-8/PP.

### 3.3. About Mechanism

**Figure 7:**
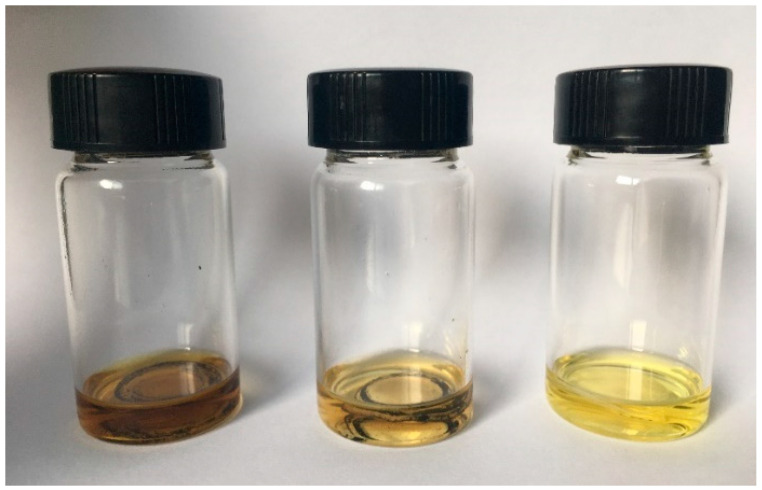
Adsorption tests of KB@ZIF-8/PP separator, KB/PP separator and PP separator.

The KB@ZIF-8/PP separator, KB/PP separator and PP separator after 100 cycles at 0.1 C were immersed in the same volume of lithium–sulfur battery electrolyte, and the adsorption of polysulfide was judged by observing the color of the solution. In Figure 7, there are KB@ZIF-8/PP, KB/PP and PP separators in the bottles from left to right. It can be observed that KB@ZIF-8/PP dissolves more polysulfide than KB/PP and PP, indicating that ZIF-8 can effectively absorb polysulfide. This can be attributed to the Lewis acid-base interaction between ZIF-8 and polysulfide, which greatly improves the adsorption efficiency of polysulfide.

**Figure 8:**
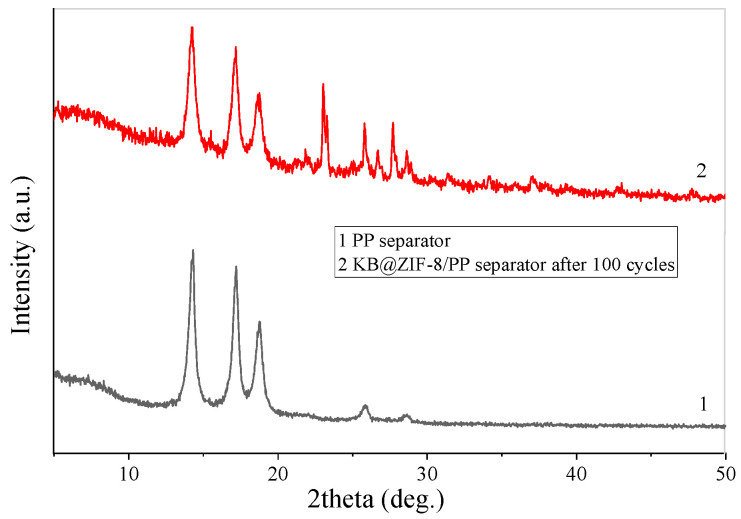
XRD patterns of KB@ZIF-8/PP separator after cycling and PP separator.

To explore whether the structure of ZIF-8 changes after cycles, XRD tests were performed on the @ZIF-8/PP separator after 100 cycles at 0.1 C and the pure PP separator. As shown in Figure 8, at the phase angles of several strong diffraction peaks of ZIF-8, there are no peaks at the KB@ZIF-8/PP after cycles. However, the diffraction peaks appear at 2θ = 23.08° and 27.75°, illustrating that the structure of ZIF-8 was destroyed, presumably because of the chemical interaction between ZIF-8 and polysulfide.

**Figure 9:**
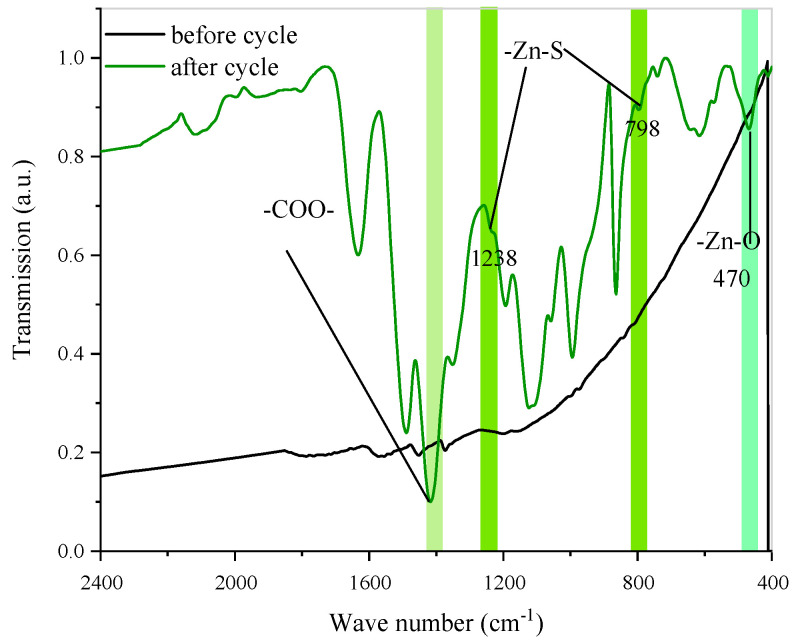
FTIR spectra of KB@ZIF-8/PP separator before and after cycling.

FTIR measurement was used to characterize the KB@ZIF-8/PP separator before the cycle and after 100 cycles at 0.1 C to confirm the chemical interaction between ZIF-8 and polysulfides. It can be seen from Figure 9 that, after cycles, interference bands corresponding to Zn-S and Zn-O appeared. ν vibration at 1238 cm^−1^ and γ vibration at 798 cm^−1^ are assigned to Zn-S, verifying the chemical interaction between ZIF-8 and polysulfide. γ vibration at about 470 cm^−1^ attributes to the Zn–O bonding, which can be explained as the adsorption of polysulfides reduces the framework flexibility of the ZIF-8.

**Figure 10:**
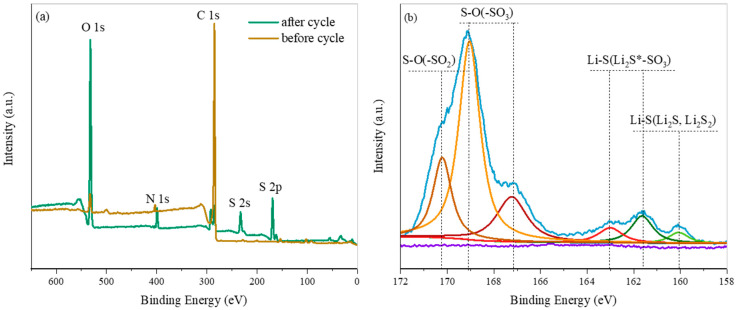
(**a**) XPS spectra and (**b**) S 2p of XPS spectra of KB@ZIF-8/PP separator before and after cycling.

To further study the chemisorption of ZIF-8, the XPS tests were performed on the KB@ZIF-8/PP separator before cycling and after 100 cycles at 0.1 C (the cell was finally discharged). As shown in Figure 10, the peaks at 285 eV, 400 eV and 530 eV can be assigned to C 1s, N 1s and O 1s, respectively. It can be observed that an obvious sulfur signal appears in the KB@ZIF-8/PP separator after cycles, but not in the separator before the cycle. The peaks at 233 eV and 161 eV correspond to S 2s and S 2p, respectively, further proving that ZIF-8 reacts with polysulfides. On the S 2p spectrum, the peaks at around 160.1 eV and 161.7 eV correspond to Li_2_S and Li_2_S_2_ of Li–S bonding, respectively, and the peaks at 161.7 eV and 163.0 eV can be assigned to Li_2_S*-SO_3_. The peaks located at around 167.2 eV and 169.1 eV are assigned to –SO_3_ of S–O bonding, and 170.3 eV is attributed to –SO_2_, respectively. The above results prove that ZIF-8 captures polysulfides through strong chemisorption.

## 4. Conclusions

In summary, a KB@ZIF-8/PP separator was prepared by vacuum filtration method and used in a lithium–sulfur battery. ZIF-8 has a high porosity and a large specific surface area, which is conducive to the adsorption of polysulfides. As an acidic material, ZIF-8 has many unsaturated metal sites, which can interact with polysulfides, thereby improving the adsorption efficiency. KB can make up for the lack of conductivity of ZIF-8, and its pore structure can play a role in physically trapping polysulfides. Therefore, the LSBs assembled with the KB@ZIF-8/PP separator show excellent electrochemical performance. At a current density of 0.1 C, they exhibit small polarization and deliver a high initial capacity of 1236 mAh/g and a relatively high capacity retention rate of 59.27% after 100 cycles. These results suggest that the KB@ZIF-8/PP separator is promising for enhancing the electrochemical performance of LSBs.

## Data Availability

Not applicable.
